# Associated Factors of Drinking Prior to Recognising Pregnancy and Risky Drinking among New Zealand Women Aged 18 to 35 Years

**DOI:** 10.3390/ijerph16101822

**Published:** 2019-05-23

**Authors:** Sherly Parackal, Mathew Parackal, John Harraway

**Affiliations:** 1 Department of Preventive and Social Medicine, University of Otago, Dunedin 9016, New Zealand; 2 Department of Marketing, University of Otago, Dunedin 9016, New Zealand; mathew.parackal@otago.ac.nz; 3 Department of Mathematics and Statistics, University of Otago, Dunedin 9016, New Zealand; jharraway@maths.otago.ac.nz

**Keywords:** drinking prior to recognising pregnancy, contraception use, risky drinkers, smoking, alcohol exposed pregnancy, New Zealand

## Abstract

Nearly half of all pregnant women in the Western world drink prior to recognising pregnancy. The current study aimed to investigate the factors associated with drinking prior to recognising pregnancy among pregnant women and factors associated with risky drinking among nonpregnant sexually active women. The study was a cross-sectional survey of a random sample of women aged 18 to 35 years (*n* = 1062) selected from the New Zealand electoral roll. Pregnant women (currently pregnant: *n* = 65; previously pregnant: *n* = 202) who were risky drinkers and who smoked in the year prior to pregnancy had five times the odds (*p* < 0.01) and women who planned their pregnancy (*p* = 0.05) and who used a community service card (*p* = 0.004) had less than half the odds to drink prior to recognising pregnancy than their respective counterparts. Among sexually active nonpregnant women who consumed alcohol, those who smoked in the year prior to the survey and those who drank for social reasons, for mood enhancement or coping reasons had higher odds of being risky drinkers (*p* < 0.05). Addressing risky drinking, especially in social settings, and smoking among women of peak childbearing age may mitigate the potential risk of drinking prior to recognising pregnancy.

## 1. Introduction

Foetal alcohol spectrum disorder (FASD), a consequence of maternal drinking is prevalent in 3–5% of children in the United States [1] and in about 8 per 1000 children and youth on average globally [2]. Observations from epidemiological studies indicate that the majority of women who drink in pregnancy do so prior to recognising pregnancy [3,4]. Given that early pregnancy is a vulnerable period for alcohol teratogenicity [5], current government guidelines in countries such as New Zealand [6], Australia [7], Canada [8], USA [9] and the UK [10] include a recommendation of abstinence when planning a pregnancy or thinking one could become pregnant. Despite these guidelines, drinking in the period prior to recognising pregnancy is widely prevalent. 

The findings from the New Zealand Alcohol in Pregnancy Study, a national cross-sectional survey of a representative sample of women aged 16 to 40 years showed that the majority of women who drank in pregnancy (~50%) stopped drinking on pregnancy recognition (77% of those who drank any alcohol during pregnancy) but prior to pregnancy recognition drank heavily [3]. Similar levels of drinking in early pregnancy have been reported in the US (45%) [4] and Canada (50%) [11] but higher levels in Australia (60%) [12] and Ireland (81%) [13]. The prevalence of binge-drinking before pregnancy recognition is reported to be 18–20% in Australia [14,15], 25% in Denmark [16], 13% in Canada [17] and 17% in New Zealand [3].

One reason for a higher proportion of women drinking before recognising pregnancy in comparison to those consciously drinking in pregnancy may be that they did not expect to become pregnant. Studies have shown that significantly lower proportions of women with unintended pregnancies, both unwanted (37%) as well as mistimed (39%), recognised pregnancy in the first month in comparison to women who intended to become pregnant (58%) [18]. Non- or irregular use of contraception [19] can lead to unintended pregnancies, which is estimated to be about 37% of all live births in the US [20]. Among women with unwanted pregnancies in the US, 56% had consumed alcohol in the month prior to recognising pregnancy and 23% had binged during this period [21]. Similar results were reported by Terplan et al. [22], where women with unwanted pregnancies were more likely to binge-drink than women with intended or mistimed pregnancies in the three months prior to pregnancy. However, no differences in heavy drinking (seven or more drinks in a week) based on pregnancy intentions were observed between the two groups of women [22]. 

Pregnancy planning has been found to be associated with drinking patterns but not with cessation of drinking. In a Swedish study that investigated how women planned their pregnancies, only 10% of women changed their alcohol consumption patterns during the planning period [23]. A Danish study that used a standardised scale to capture the various levels of pregnancy planning showed that there were no differences in the prevalence of binge-drinking in early pregnancy among women with a high degree of planning in comparison to those with a low degree of pregnancy planning (20% vs. 31%) [24]. In contrast to information-seeking and intake of folic acid, changing alcohol consumption was not a pregnancy planning behaviour [25].

In the study by Pryor and colleagues, overall alcohol use was similar among women with planned/intended (55%) and unintended pregnancies (56%); however, women with planned/intended pregnancies were less likely to binge (19% vs. 28%) in early pregnancy in comparison to women with unintended pregnancies [26]. Other studies have echoed this finding, showing that similar proportions of women with or without planned pregnancies continue to drink in the early stages of pregnancy (47% vs. 53%); however, those with planned pregnancies drink significantly lower amounts of alcohol per occasion (1.98 vs. 2.74 drinks) [11]. In the study by Tough and colleagues [11], overall drinking patterns were similar during the prerecognition period but changed after pregnancy was recognised [11]. 

As a high proportion of usually drinking women of childbearing age continue to drink prior to recognising pregnancy, addressing and monitoring this behaviour is of paramount significance to prevent alcohol-exposed pregnancy and, hence, FASD. In New Zealand, the first Alcohol in Pregnancy study last collected data on drinking prior to pregnancy recognition in 2005 [3], and hence current information on the prevalence and associated factors of drinking prior to recognising pregnancy was urgently needed. Furthermore, studies have also shown that risky drinking patterns prior to pregnancy are a significant predictor of drinking prior to recognising pregnancy [3,27]. However, there is a dearth in studies that have identified factors associated with risky drinking among sexually active women to facilitate targeted interventions to reduce the risk of drinking prior to recognition of pregnancy. Hence, the current study aimed to investigate the factors associated with (1) drinking prior to recognising pregnancy among pregnant women and (2) risky drinking among nonpregnant sexually active women. The demographic make-up, alcohol and tobacco consumption and knowledge of guidelines on drinking in pregnancy are also reported for women of different maternal status. In addition, data are also reported on types of drinkers in pregnancy (pregnant women), frequency of contraception use, drinking motives and drinking refusal self-efficacy scores and level of agreement on outcomes of maternal drinking of sexually active nonpregnant women who consumed alcohol.

## 2. Materials and Methods

### 2.1. Study Design

The aims of the study were achieved using a cross-sectional survey design. A hybrid survey approach was used [28] to facilitate participation either via mail or the World Wide Web. Based on the evidence that younger women have a higher level of risk of drinking before recognising pregnancy in New Zealand [3] and the changing demographic trends in women’s fertility in New Zealand showing that the peak childbearing age has shifted upwards to 35 years [29], the target population studied was women aged 18 to 35 years. The study received ethics approval from the University of Otago Human Ethics Committee on 25 November 2015 (Ref 15/154). 

### 2.2. Sample Size Calculation and Participant Selection

The study used a simple random sampling method to select the names and addresses of women who met the age criteria from the New Zealand Electoral Roll in December 2015. In general, the response rate to survey research is low, particularly for younger people in New Zealand recruited using the electoral roll [30]. Hence, 3250 women were contacted to obtain a final sample size of 1100 women (anticipated response rate of 35%) to report results within 3% error. The women were contacted via their postal address using a letter to solicit participation. The mail-out included the invitation letter, participant information sheet, questionnaire and a reply-paid envelope. 

### 2.3. Data Collection

Data collection was achieved via a pretested questionnaire specifically designed to meet the objectives of the study. The questionnaire was piloted on a sample of thirty-one women meeting the inclusion criteria set for the study. The purpose of the pilot study was to estimate the time taken to complete the questionnaire and identify any issues regarding question wording. Participants could choose to take part in the survey either by sending back the completed questionnaire using the freepost envelope enclosed or by logging into the password-protected site using the personalised login details provided in the invitation letter. A reminder postcard was sent to those who did not respond after two weeks of the first mail out (survey wave 2). Following this, a reminder letter with the questionnaire and a reply-paid envelope were mailed out to those who had still not responded (survey wave 3). 

### 2.4. Maternal Status

**Figure 1:**
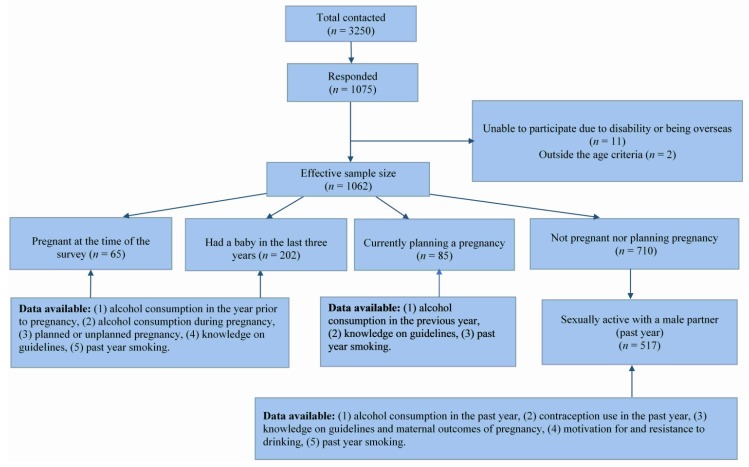
Schematic representation of the study sample and data available according to maternal status.

Responses to the question that asked participants’ current maternal status were used as a skip question to direct them to different sets of questions (Figure 1). Women who were pregnant at the time of the survey (Currently pregnant; *n* = 65) and women who had had a baby in the three years preceding the survey (Previously pregnant; *n* = 202) were directed to questions on alcohol consumption behaviours during pregnancy, alcohol consumption in the year prior to pregnancy and knowledge about current guidelines on drinking in pregnancy. Data from these women were used to achieve the first objective of the study. 

Women who indicated that they were currently planning a pregnancy (planning pregnancy; *n* = 85) were directed to questions on alcohol consumption behaviours and knowledge about current guidelines on drinking in pregnancy. It is important to mention that the option provided was “currently planning a pregnancy” and there is a high likelihood that there was no uniformity among this group in interpreting what “current” meant. 

Women who indicated that they were not currently pregnant nor had a baby in the past three years nor were currently planning a pregnancy (nonpregnant; *n* = 710) were directed to questions on sexual behaviour, contraception use, level of agreement on outcomes of maternal drinking, knowledge about guidelines on drinking in pregnancy, past year alcohol and tobacco consumption and questions on motivation for and resistance to alcohol consumption. Among the nonpregnant women, 517 were sexually active with a male partner, and data from these women were used to achieve the second objective of the study. 

### 2.5. Demographic Measures

Age data were collected using the “year of birth” question, and any missing data were replaced by the age recorded on the electoral roll (*n* = 42). Data on ethnicity, the level of education, household income, employment status and marital status were collected using standardised questions from the New Zealand 2013 Census [31]. From the ethnicity data, prioritised ethnicity was determined in the order of Māori > Pacific > Asian > New Zealand European/Other (NZEO). Due to a low number of Pacific women in the sample, Māori and Pacific ethnic categories were combined, as women of these two ethnic groups have similar prevalence of hazardous and binge-drinking in contrast to New Zealand European and Asian ethnicity [32]. The level of education data was re-coded as “No tertiary education” and “Some or completed tertiary education”. Annual household income categories were collapsed to create four categories, namely, “Less than 30,000”, “30,000 to 70,000”, “More than 70,000” and “Prefer not to answer or don’t know”. Data were also collected on whether participants used a community service card, an indicator of socioeconomic deprivation, using yes/no options which were re-coded as 1 and 0, respectively. Due to a high proportion of missing or “don’t want to answer” data points for the household income variable, the use of the community service card, a surrogate measure of deprived socioeconomic status in New Zealand [33], was used in all the analyses. Data on marital status were collapsed to form two categories, namely, “In a permanent relationship” and “Not in a permanent relationship”. 

### 2.6. Knowledge Measures

Data on participants’ level of agreement with statements on outcomes of maternal drinking were collected on a 7-point Likert scale (1 = Strongly Disagree and 7 = Strongly Agree) only from nonpregnant women (*n* = 710) using the following seven statements: (1) A baby is particularly vulnerable to harms from alcohol during the early stages of pregnancy, i.e., the first 8 to 10 weeks of pregnancy. (2) If you have drunk some alcohol while you are pregnant, stopping at any time is good for your baby. (3) Drinking 1 or 2 drinks once or twice a week is okay when you are pregnant. (4) Not drinking any alcohol at all during pregnancy is best for the baby. (5) When a pregnant women drinks, alcohol can pass through the placenta to the baby. (6) Drinking alcohol at any time during pregnancy can harm the baby. (7) Drinking alcohol during pregnancy can lead to life-long disabilities in a child. A “Don’t Know” option was also provided and coded as zero. First, the scores of the negative statement (3) was reverse-scored, and then a composite mean score (total score divided by 7) was calculated and used as a continuous variable. 

Data on knowledge about the guideline on alcohol consumption during pregnancy were collected using yes/no and re-coded as 1 and 0, respectively, from all women. 

### 2.7. Contraception Measures

Contraception data were collected using the following questions. (1) Are you now or ever been sexually active? Yes/No (2) If you answered YES to the previous question, have you had sex with a male partner in the last 12 months, Yes/No. (3) When you had sex with a male partner in the last 12 months, did you use any contraception (including vasectomy or tubal ligation). Response options were: Always, Sometimes and Never.

### 2.8. Consumption Measures

Data on alcohol consumption in the 12 months before the survey (Nonpregnant women), 12 months prior to the current or previous pregnancy (Pregnant women) and during pregnancy (current or previous; Pregnant women) were collected using the three consumption questions of the Alcohol Use Disorders Identification Test (AUDIT-C) [34], as this instrument has been shown to have high sensitivity (95%) and specificity (85%) to identify risky drinking [35], a key variable of interest in the current study. The New Zealand standard drink definition was provided both in the written form as well as in the graphic form to enable participants to provide data on the number of standard drinks consumed. This definition read as:

“A 330 mL bottle/stubby or can of normal strength beer or a 30 mL measure of spirits mixed or straight, or 1 can of ready to drink (RTD) contains around one standard drink. 100 mL of wine is one standard drink, so a small 150 mL glass of wine contains one and a half standard drinks, a medium 200 mL wine glass contains two standard drinks and a typical 750 mL bottle of wine contains around eight standard drinks.”

Information on motives for drinking in various situations was collected using Coopers Drinking Motives Questionnaire—Revised (DMQR) [36] only from women who were nonpregnant (*n* = 710). The DMQR contains 20 reasons people might be motivated to consume alcohol. Participants rate on a 5-point scale (1 = Almost Never/Never; 5 = Almost Always/Always) how often they would be motivated to drink for the listed reasons. These listed reasons were then reduced to four dimensions (five items each; maximum score for each dimension = 25), namely, social motives, coping motives, enhancement motives and conformity motives [36].

The Drinking Refusal Self-Efficacy Questionnaire—Revised (DRSEQ-R) [37] was used to assess the ability of women who were nonpregnant (*n* = 710) to resist alcohol in different contexts. This scale has 19 items against which participants are asked to rate their ability to resist alcohol in various situations on a 6-point scale (1 = I am very sure I could not resist drinking; 6 = I am very sure I could resist drinking). The responses were then reduced to three dimensions, namely, social pressure (five items; maximum score = 30), emotional relief (seven items; maximum score = 42) and opportunistic (seven items; maximum score = 42). Higher scores indicate a greater level of confidence in resisting drinking due to social pressure, for emotional relief and opportunistic drinking [37].

Data on smoking status were collected for the 12 months prior to the survey by asking all participants whether they smoked or not (never smoked and smoked) in the 12 months preceding the survey or 12 months prior to their current or previous pregnancy. Those who identified themselves as smokers (both occasional and regular) were coded as “1” and nonsmokers as “0”. 

### 2.9. Factors Associated with Drinking Prior to Recognising Pregnancy among Pregnant Women

To identify types of drinkers in pregnancy, both currently (*n* = 62) and previously (*n* = 201) pregnant women were asked to tick one of the following options: (1) I had some alcohol but only before I knew I was pregnant and stopped; (2) I had some alcohol before I knew I was pregnant and continued; (3) I drink/drank being aware of my pregnancy; (4) I stopped drinking alcohol before I became pregnant; (5) Not sure; (6) I don’t drink alcohol anyway. Women who chose either the first or second option were combined to form the response category “drank prior to recognising pregnancy” (*n* = 263), as this was the key question of interest to the study. The explanatory variables of interest were (1) risky drinking prior to pregnancy, (2) planned pregnancy (yes/no), (3) smoked prior to pregnancy, (4) age, (5) ethnicity, (6) marital status, (7) education, (8) employment, (9) use of community service card and (10) knowledge of guidelines on alcohol consumption during pregnancy. 

### 2.10. Factors Associated with Risky Drinking among Sexually Active Nonpregnant Women Who Consumed Alcohol

Risky drinking was defined as an AUDIT-C score of 3 and above [38]. The explanatory variables of interest identified were (1) participants’ level of agreement with statements on outcomes of maternal drinking, (2) knowledge about guidelines on alcohol consumption in pregnancy, (3) frequency of contraception use, (4) smoking status, (5) motivation for drinking, (6) resistance to drinking, (7) age, (8) ethnicity, (9) level of education, (10) employment status, (11) use of community service card and (12) marital status. Composite scores of participants’ level of agreement with statements on outcomes of maternal drinking, the four dimensions of motivation for drinking and the three dimensions of the resistance to drinking in various situations were included as continuous variables in the analysis. Separate analyses were done, with abstainers being included and excluded. 

### 2.11. Analysis

Survey (post-stratification) weights were calculated for the 1062 valid responses based on the 2013 Census data for women by ethnicity (detailed ethnicity groups) and age group (15–19, 20–24, 25–29, 30–34, 35–39). Prioritised ethnicity was determined for each ethnicity category in the Census to match that used here (i.e., Māori, Pacific, Asian, and finally European/Other) using frequencies for single, double and triple ethnic groups from the Census and based on counts estimated for the age groups used here (i.e., 18–24, 25–29, 30–34) using percentages of the relevant census age groups as necessary. Respondents without age data were given the mean weight of their ethnic group; for respondents without an ethnic group, the same was done using their age group; and for those lacking both, the mean weight was used. Percentages (95% CI) and medians (Inter Quartile Range (IQRs)) were calculated for various variables broken down by pre-specified strata, and these analyses incorporated the survey weights. Descriptive statistics were used to report the demographic makeup, frequency of contraception use, consumption measures, awareness of guidelines on drinking in pregnancy, types of drinkers in pregnancy, participants’ level of agreement with statements on outcomes of maternal drinking, drinking motives and drinking refusal self-efficacy scores. Binary logistic regression was used to investigate the factors associated with “drinking prior to recognising pregnancy” (among pregnant women) and “risky drinking” (nonpregnant sexually active women), the outcomes of interest of the study. A backward stepwise elimination procedure was used to identify the best model. All statistical analyses were conducted using Stata 14.1 (StataCorp LP, College Station, TX, USA) and SPSS VS 24 (IBM Corp, Armonk, NY, USA) with two-sided *p* < 0.05 considered statistically significant. 

## 3. Results

The survey obtained a response rate of 37% (*n* = 1062) after adjusting for address accuracy of the electoral roll (0.98), undelivered mail (*n* = 311), inability to participate due to disability issues (*n* = 11) and wrong age group (<18, >35 years; *n* = 2). The majority of women (81%; *n* = 864) participated using the paper version of the questionnaire. 

### 3.1. Demographic Characteristics, Alcohol and Tobacco Consumption and Knowledge of Alcohol in Pregnancy Guidelines According to Maternal Status

The findings of our study indicate that in our sample, 5.2% (95% CI 4.0–6.8) of women were pregnant at the time of the survey (Currently Pregnant), 18.4% (95% CI 16.0–21.1) had a baby in the three years preceding the survey (Previously pregnant), 8.7% (95% CI 6.9–10.9) were planning a pregnancy at the time of the survey (Planning pregnancy) and 67.6% (95% CI 64.5–70.6) were none of the above (Nonpregnant) (Table 1). 

NZEO women were overrepresented across all the maternal status categories (*p* = 0.002; Table 1). Women aged 25 years and over were more likely to be currently pregnant and planning a pregnancy in comparison to the younger age; however, women in oldest age group (30–34 years) were more likely to have had a baby in the three years preceding the survey (Previously pregnant) in comparison to the younger age groups (*p* < 0.001; Table 1). Women in a permanent relationship and women who had completed university education were more likely to be either planning a pregnancy or currently or previously pregnant (*p* < 0.05; Table 1). Women who were previously pregnant were less likely to be employed (*p* < 0.001; Table 1). Use of community service card, an indicator of low socioeconomic status, was similar among women of different maternal status (*p* = 0.294; Table 1). Overall, a high proportion of women were aware of the government guidelines on alcohol consumption during pregnancy; however, these women were more likely to be either currently or previously pregnant in comparison to women who were not pregnant and those planning pregnancy (*p* = 0.018) (Table 1).

### 3.2. Pregnant Women

#### 3.2.1. Types of Drinkers in Pregnancy

There were no statistically significant differences in the response options of currently pregnant and previously pregnant women (*p* = 0.546; Table 2). About 50% (95% CI 43.5–56.9) of pregnant women stopped on recognising pregnancy and 10% (95% CI 6.7–13.7) who drank prior to recognising pregnancy continued to drink in pregnancy (Table 2). About 75.4 % (95% CI 62.0–85.2) of currently pregnant women and 64.1% (95% CI 56.1–71.4) of previously pregnant women planned their pregnancy, and there was no difference in the proportion of currently and previously pregnant women who planned their pregnancy (*p* = 0.137) (data not shown).

#### 3.2.2. Factors Associated with Drinking Prior to Pregnancy Recognition among Pregnant Women

The backwards stepwise logistic regression analysis was performed to investigate the factors associated with drinking prior to recognition of pregnancy, and the Hosmer and Lemeshow test confirmed good fit to the data. Women who were “risky drinkers” in the year prior to pregnancy were five times more likely to drink prior to recognising pregnancy than women who were not risky drinkers (*p* < 0.001; Table 3). Similarly, women who smoked in the year prior to pregnancy also had five times higher odds of drinking prior to recognising pregnancy, albeit a wide confidence interval of the odds ratio (1.51–20.11; Table 3). For women who planned their pregnancy, the odds of drinking prior to pregnancy recognition were halved compared to women who had not planned their pregnancy (odds ratio = 0.47; 95% CI of odds 0.22–1.00; *p* = 0.052; Table 3). Similarly, women who were of low income and used a community service card were also less likely to drink prior to recognising pregnancy (odds ratio 0.28; 95% CI 0.12–0.67; *p* = 0.004). Age, ethnicity, level of education, employment status, marital status or knowledge of guidelines on alcohol consumption in pregnancy were not significant factors associated with drinking prior to recognising pregnancy.

### 3.3. Nonpregnant Women

#### 3.3.1. Drinking Motives and Drinking Refusal Self-Efficacy Scores of Nonpregnant Sexually Active Women

Data on motivation for drinking and drinking refusal self-efficacy are only reported for women who were nonpregnant and sexually active with a male partner (*n* = 517). Drinking for social reasons had the highest mean (14.1; SD 5.2) and median (14) scores compared to the coping, enhancement and conformity domains (Table 4). Consequently, the mean (24.5; SD 5.5) and median (26) drinking refusal self-efficacy score were lowest for the social pressure domain in comparison to the other two domains (Table 4).

#### 3.3.2. Participants’ Level of Agreement on Outcomes of Maternal Drinking

As seen in Table 5, the mean scores of the level of agreement with all the seven statements were at the high end of the scale, indicating a high level of awareness on outcomes of drinking during pregnancy in this sample of sexually active women.

#### 3.3.3. Factors Associated with Risky Drinking among Nonpregnant Sexually Active Women Who Consumed Alcohol

The model reported here was developed using backwards stepwise procedures with the Hosmer and Lemeshow Test, confirming good fit to the data. The results of the binary logistic regression analysis are reported in Table 6. The findings indicate that women of Asian ethnicity had lower odds of being risky drinkers in comparison to New Zealand European women (OR = 0.08; 95% CI (0.03, 0.25); *p* < 0.001). Women who smoked in the year prior to the survey (OR= 2.70; 95% CI (1.14–6.28); *p* = 0.025) and who drank for social (OR = 1.09; 95% CI (1.01, 1.18); *p* = 0.02), for mood enhancement (OR = 1.23; 95% CI (1.11, 1.36); *p* < 0.001) and coping reasons (OR = 1.24; 95% CI (1.08, 1.44); *p* = 0.003) had higher odds of being risky drinkers (Table 6). By contrast, women who drank for conformity reasons did not have higher odds of being risky drinkers (*p* > 0.05). Other demographic variables, knowledge of alcohol consumption guidelines during pregnancy and while planning a pregnancy, participants’ level of agreement with statements on outcomes of maternal drinking and drinking refusal self-efficacy scores did not predict risky drinking (*p* > 0.05).

## 4. Discussion

The salient findings of the current study are that the majority of pregnant women who consume alcohol in pregnancy do so prior to recognising pregnancy with risky drinking patterns prior to pregnancy being a significant contributing factor. In order to facilitate addressing drinking prior to recognising pregnancy, the current study also aimed to identify factors associated with risky drinking among sexually active nonpregnant women. Unpacking drinking behaviour further using motivation for drinking and drinking refusal self-efficacy scores indicates that risky drinkers had higher odds to drink for social reasons, for enhancing positive moods and for coping reasons. Smoking was significantly associated with drinking prior to recognition of pregnancy among pregnant women as well as risky drinking among nonpregnant sexually active women.

About 75% of pregnant women in the current study had planned their pregnancy, which is similar to that reported by McCormack et al. [12] and Pyror et al. [26]. In the current study, women with planned pregnancies were less likely to drink prior to recognition of pregnancy (odds ratio = 0.47; 95% CI of odds 0.22–1.01; *p* = 0.052; Table 3), which is similar to that reported by McCormack et al. [12].

Interestingly, knowledge about guidelines on alcohol consumption in pregnancy was not a protective factor for women drinking prior to recognising pregnancy. Although we found no other studies to compare our findings, the results of an initiative to improve knowledge, attitudes and behaviour regarding alcohol consumption in pregnancy observed an improved level of knowledge regarding the effects of drinking in pregnancy in the intervention group but no statistically significant difference in drinking behaviour during pregnancy between the intervention and control group [39]. These observations seem to indicate that depending on dissemination of knowledge alone to prevent alcohol-exposed pregnancies may not be prudent.

The observation that risky drinking prior to pregnancy among pregnant women is a significant predictor of drinking prior to recognising pregnancy corroborates previous findings [3,13,16,17,27]. Hence, investigating the factors associated with risky drinking among nonpregnant sexually active women is a pragmatic approach to mitigate the potential risk of drinking prior to recognising pregnancy. Our findings clearly show that women who were motivated to drink for social reasons, mood enhancement and for coping were more likely to be risky drinkers in contrast to women who drank for the sake of conformity (Table 6). These findings provide the impetus to further research drinking motivations to enable targeted interventions to reduce risky drinking. Further analyses of our data (results not shown) indicate that women aged 18 to 24 years and 25 to 29 years had similar scores for motivation for social drinking (*p* = 0.076), motivation for mood enhancement (*p* = 0.086) and motivation for coping reasons (*p* = 0.828), but both these age groups had higher mean scores than women aged 30–35 years (*p* < 0.05) for all the three domains that were significant predictors of risky drinking (Table 6). Findings from our previous study showed that pregnant women aged 30 years or younger had a higher risk of drinking prior to recognising pregnancy [3], which is similar to that observed among nonpregnant sexually active women in our current study. Frequency of contraception use was not a significant predictor of risky drinking. Nevertheless, 21% (data not shown) of women who consumed alcohol indicated that they used contraceptives “sometimes” or “never”. This is a concern, as non- or irregular use of contraception can lead to unintended pregnancies [19] and, hence, a higher risk of drinking prior to recognising pregnancy [21].

Despite the wide confidence interval of the odds, smoking was also a significant predictor of drinking prior to recognising pregnancy among pregnant women and of risky drinking among nonpregnant sexually active women. Similar findings have been reported by Floyd et al. [4] in the US, Starndberg-Larsen et al. [16] in Denmark and O’Connor et al. [40] in South Africa. In the study by Starndberg-Larsen et al., smoking predicted binge-drinking in both the unrecognised and recognised part of pregnancy [16]. In the study by O’Connor et al., smoking was a risk factor for frequent drinking prior to recognising pregnancy [40].

The strength of the current study is the national scope. Nevertheless, the study has several limitations. Firstly, the somewhat lower response rate (nonresponse bias) achieved for the study can limit the extrapolation of the findings to the target population, although it reflects current trends in survey research. For example, a recent Canadian study that aimed to compare response rates to different survey modes and survey incentives reported a total response of 28% with the highest response (43%) being for a short-mailed questionnaire [41]. The response rate of 37% received for the current study is comparable to the Canadian study [41], although the participants of the latter study were much older (mean 57.3 years SD 17.1) than those of the current study (mean 25.4 years; SD 4.5). Other biases such as selection bias, for, e.g., exclusion of those not on the electoral roll cannot be overruled. The lower response rate may also be due to using the electoral roll as a sampling frame, as people of younger age sampled from the electoral roll have been shown to be less likely to respond [30]. Hence, over 3000 women were contacted to enable achieving an adequate sample size for the study. Despite these limitations, using the electoral roll as a sampling frame is advantageous in that it has high coverage and provides additional information, such as region and deprivation index. The continuum of resistance model [42] is one framework used to assess nonresponse bias. According to this model, the late responders are most similar to the nonresponders [42]. Hence in the current study, associations between response latency and key demographic and behavioural variables were investigated. The findings of this analysis indicated that there was no significant association between response latency and age (*p* = 0.269), ethnicity (*p* = 0.059), deprivation index (*p* = 0.317), region (*p* = 0.699), employment (*p* = 0.494), household income (*p* = 0.751), past year smoking (*p* = 0.8333), risky drinking (nonpregnant; *p* = 0.469), risky drinking prior to pregnancy (pregnant; *p* = 0.672) and maternal status (*p* = 0.498). Some differences were found in response latency according to education, with a lower percentage of those with some/completed secondary school education responding in wave 2 in comparison to waves 1 and 3 (*p* = 0.028). However, this observation is unlikely to have any significant impact on the survey estimates because, according to the response latency model, late responders are more similar to nonresponders [42]. The lower response by those with some/completed secondary school education in wave 2 was probably because in wave 2, only a reminder postcard was sent in contrast to including the hard copy of the questionnaire in waves 1 and 3. These findings give us the confidence that the nonresponders are unlikely to be different to those who responded to the survey in their alcohol consumption behaviour or their demographic characteristics.

The proportion of pregnant women in the current study (5.2% 95% CI 4.0–6.8; Table 1) was slightly lower than that in the population of similar aged women (20–34 years) which is approximately 7% of live births [43] and 1% foetal or infant deaths [44]. Further, recall bias, especially among women who were reporting alcohol consumption during a previous pregnancy, also cannot be overruled. Nevertheless, the weighted estimates of prevalence of drinking in pregnancy in the current study for women who were currently pregnant and those who were pregnant in the three years prior to the survey was similar (*p* = 0.546; Table 2). These estimates were also similar to that previously done using a more robust methodology [3], giving us confidence in the survey estimates reported.

Smoking data were collected using a dichotomous response option which resulted in not capturing differences between regular and occasional or social smokers. Although in hindsight, it would have been prudent to collect these data, the total proportion of smokers in pregnancy both among currently pregnant women (15.3; 95% CI 7.9–27.7; Table 1) and previously pregnant (18.1; 95% CI 13.0–24.6) women were similar to that reported in the population (14.2%) for 2015 [43], giving confidence in the findings of the study.

Respondent bias due to the self-reported nature of the data may have also impacted the findings of the study. For example, questions on knowledge about consequences of maternal drinking were asked in tandem with alcohol consumption behaviour questions; hence, the response to one could have been biased by the response to the other. Further, as the population of interest of the study was women aged 18 to 35 years, these findings cannot be extrapolated to women over this age who are in their childbearing years.

Overall, the findings of the current study are particularly important as the majority of women who drink in pregnancy do so in the early stages or the first trimester due to an unintended [17,27] or delayed recognition of pregnancy [3,13,16]. At the population level, the proportion of women consciously drinking in pregnancy, including those who are heavy drinkers, is a much smaller proportion, about 9% [45] in comparison to those who drink prior to recognising pregnancy (~50%) [3,4,11,12,19]. Most studies collecting data on alcohol consumption in pregnancy either retrospectively or at one time point report a much lower prevalence, in contrast to studies that assess alcohol consumption at different time points in pregnancy [45] or specifically ask if women had drunk alcohol prior to recognising pregnancy [3]. Addressing risky drinking and smoking among women of peak childbearing age would have positive impacts on their own health as well as reduce the risk of potentially drinking prior to recognising pregnancy and hence should be a high priority public health initiative targeting women of childbearing age. 

## 5. Conclusions

The findings of the current study indicate that the drivers of drinking prior to recognising pregnancy may be the same as risky drinking among nonpregnant women of peak childbearing age. Public health efforts addressing risky drinking, especially in social contexts, and smoking among women of peak childbearing years may reduce the prevalence of drinking prior to recognising pregnancy and, hence, alcohol exposed pregnancy.

## Figures and Tables

**Table 1:** Demographic characteristics, alcohol and tobacco consumption and knowledge of alcohol in pregnancy guidelines according to maternal status (*n* = 1062) ^1^.

	Maternal Status % [95% CI]			
	Nonpregnant *N* = 710	Planning Pregnancy *N* = 85	Previously Pregnant *N* = 202	Currently Pregnant *N* = 65
**Total**	67.6 [64.5–70.6]	8.7 [6.9–10.9]	18.4 [16.0–21.1]	5.2 [4.0–6.8]
**Age Category (years)**				
18–24	57.0 [53.1–60.9]	21.6 [13.0–33.7]	12.1 [7.6–18.9]	9.2 [3.8–20.7]
25–29	24.9 [21.7–28.3]	37.2 [26.2–49.6]	30.1 [23.4–37.8]	45.6 [33.1–58.8]
30–34	18.1 [15.4–21.1]	41.2 [30.5–52.9]	57.8 [50.0–65.2]	45.2 [32.7–58.3]
Chi ^2^ = 206.1413; *p* < 0.001				
**Prioritised Ethnicity ^2^**				
Māori	17.9 [14.9–21.3]	22.7 [14.5–33.7]	14.9 [10.3–20.9]	10.1 [4.2–22.2]
Pacific	5.3 [3.1–8.7]	20.6 [10.7–35.8]	11.4 [6.2–20.2]	3.0 [0.4–18.3]
Asian	17.0 [13.6–21.1]	16.2 [8.9–27.6]	18.9 [13.1–26.4]	19.9 [10.0–35.5]
New Zealand European/other	59.9 [55.5–64.0]	40.5 [30.2–51.8]	54.8 [47.0–62.5]	67.1 [52.2–79.2]
Chi ^2^ = 41.4504; *p* = 0.002				
**Marital Status ^3^**				
Single/widowed/divorced	68.2 [64.4–71.7]	24.5 [15.1–37.1]	14.2 [9.6–20.5]	18.2 [9.8–31.2]
Permanent relationship	31.8 [28.3–35.6]	75.5 [62.9–84.9]	85.8 [79.5–90.4]	81.8 [68.8–90.2]
Chi ^2^ = 235.0809; *p* < 0.001				
**Highest level of Education ^4^**				
No secondary education	1.0 [0.4–2.3]	2.0 [0.5–7.8]	3.8 [1.8–7.9]	2.0 [0.3–12.7]
Some/Completed secondary education	25.0 [21.4–28.8]	20.3 [12.0–32.5]	17.7 [12.5–24.5]	17.8 [9.9–29.9]
Some University	29.5 [25.9–33.3]	22.0 [13.4–33.9]	23.5 [17.7–30.5]	17.0 [8.7–30.5]
Completed University	44.6 [40.6–48.6]	55.7 [43.4–67.3]	55.0 [47.4–62.4]	63.2 [49.5–75.1]
Chi ^2^ = 24.0947; *p* = 0.015				
**Current Employment ^5^**				
Employed	77.0 [73.3–80.3]	75.8 [64.1–84.6]	60.3 [52.8–67.4]	83.4 [70.1–91.5]
Unemployed	23.0 [19.7–26.7]	24.2 [15.4–35.9]	39.7 [32.6–47.2]	16.6 [8.5–29.9]
Chi ^2^ = 24.7187; *p* < 0.001				
**Use of Community Service Card ^6^**				
Yes	19.7 [16.9–22.8]	16.5 [8.6–24.4]	20.8 [15.2–26.4]	10.8 [3.2–18.3]
Chi ^2^ = 3.716; *p* = 0.294				
**Consumption Measures**				
Risky Drinking (AUDIT-C ≥ 3) ^7^ YES	61.0 [56.9–65.0]	56.4 [44.2–67.9]	55.6 ^8^ [47.5–63.4]	56.0 ^7^ [42.2–68.9]
Chi ^2^ = 4.668; *p* = 0.198				
Smoked YES	23.2 [20.0–26.8]	34.5 [24.1–46.7]	18.1 ^7^ [13.0–24.6]	15.3 ^7^ [7.9–27.7]
Chi ^2^ = 12.986; *p* = 0.005				
**Contraception use among sexually active nonpregnant women (*n* = 517)**				
Always	74.5 [69.9–78.6]	Not collected		
Sometimes	18.3 [14.8–22.4]			
Never	7.2 [4.8–10.7]			
**Knowledge measures ^9^**				
Knowledge about the government guideline on drinking in pregnancy: Pregnant women or those planning to get pregnant drink NO alcohol as there is no known safe level of alcohol use at any stage of pregnancy				
Yes	88.1 [85.7–90.5]	82.9 [74.8–90.3]	92.4 [88.7–96.1]	96.9 [92.6–99.9]
Chi ^2^ = 10.118; *p* = 0.018				

^1^ Maternal status data missing for 8 women; ^2^ missing data = 15; ^3^ missing data = 22; ^4^ missing data = 27; ^5^ missing data = 26; ^6^ missing data = 31; ^7^ Alcohol Use Disorders Identification Test-Consumption ^8^ this data was collected for the year prior to the past or current pregnancy; ^9^ missing data = 30.

**Table 2:** Alcohol consumption in pregnancy among currently pregnant women (*n* = 62) ^1^ and previously pregnant women (*n* = 201) ^2^.

Response Options	Maternal Status % [95%CI]		
	Currently Pregnant	Previously Pregnant	All Pregnant Women
I had some alcohol but only before I knew I was pregnant and stopped	46.0 [33.3–59.2]	51.5 [43.7–59.2]	50.2 [43.5–56.9]
I had some alcohol before I knew I was pregnant and continued	8.7 [3.9–18.4]	9.9 [6.6–14.7]	9.6 [6.7–13.7]
I drink/drank being aware of my pregnancy	1.4 [0.2–9.7]	2.3 [0.9–5.5]	2.1 [0.9–4.6]
I stopped drinking alcohol before I became pregnant	28.8 [18.2–42.5]	16.8 [11.7–23.5]	19.5 [14.7–25.5]
Not sure	0.0	0.9 [0.1–6.2]	0.7 [0.1–4.8]
I don’t drink alcohol anyway	15.0 [7.3–28.3]	18.6 [13.1–25.8]	17.8 [13.0–23.9]
Chi^2^ = 4.6208; *p* = 0.546			

^1^ Missing data = 3; ^2^ missing data = 1.

**Table 3:** Factors associated with drinking prior to pregnancy recognition (*n* = 265) ^1,2^.

Factor	Odds Ratio [95% CI of Odds]	*p*-Value
AUDIT-C ≥ 3 prior to pregnancy (Risky drinking)	5.20 [2.78–9.75]	<0.001
Smoke: Yes	5.51 [1.51–20.11]	0.010
Planned pregnancy: Yes	0.47 [0.22–1.01]	0.052
Use of community service card: Yes	0.28 [0.12–0.67]	0.004

^1^ Binary logistic regression; ^2^ missing = 27.

**Table 4:** Drinking motives and drinking refusal self-efficacy scores of nonpregnant sexually active women (*n* = 517).

	Minimum and Maximum Domain Scores	Mean (SD)	Median
Drinking motives			
Social ^1^	5–25	14.1 (5.2)	14
Coping ^2^	5–25	8.0 (3.3)	7
Enhancement ^3^	5–25	11.0 (4.9)	10
Conformity ^4^	5–25	6.7 (2.4)	6
Drinking Refusal Self-Efficacy			
Social Pressure ^5^	5–30	24.5 (5.5)	26
Emotional Relief ^6^	7–42	38.5 (6.0)	42
Opportunistic ^7^	7–42	40.5 (3.8)	42

^1^ Missing = 13; ^2^ missing = 8; ^3^ missing = 12; ^4^ missing = 6; ^5^ missing = 6; ^6^ missing = 6; ^7^ missing = 4.

**Table 5:** Participants’ level of agreement with statements on outcomes of maternal drinking (*n* = 517).

Knowledge Statements	Mean Score (SD)
A baby is particularly vulnerable to harm from alcohol during the early stages of pregnancy, i.e., the first 8 to 10 weeks of pregnancy	6.43 (1.2)
If you have drunk some alcohol while you are pregnant stopping at any time is good for your baby ^1^	6.10 (1.7)
Drinking 1 or 2 drinks once or twice a week is okay when you are pregnant ^2^	6.41 (1.3)
Not drinking any alcohol at all during pregnancy is best for the baby	6.72 (1.0)
When a pregnant women drinks, alcohol can pass through the placenta to the baby ^2^	6.54 (1.0)
Drinking alcohol at any time during pregnancy can harm the baby	6.44 (1.2)
Drinking alcohol during pregnancy can lead to life-long disabilities in a child	6.53 (1.0)
Composite Score	6.54 (0.7)

^1^ Missing = 2; ^2^ missing = 1.

**Table 6:** Factors associated with risky drinking among sexually active nonpregnant women who consumed alcohol ^1,2^.

Factor	Odds Ratio [95% CI of Odds]	*p*-Value
Ethnicity		
New Zealand European/Other (Referent)		
Māori or Pacific	1.25 [0.55–2.82]	0.595
Asian	0.08 [0.03–0.24]	0.000 *
Motivation for drinking		
Drinking for social reasons	1.09 [1.01–1.18]	0.021 *
Drinking for enhancement	1.23 [1.11–1.36]	0.000 *
Drinking for coping reasons	1.25 [1.08–1.44	0.003 *
Smoking ^3^		
Yes	2.70 [1.14–6.28]	0.025 *

^1^ Binary logistic regression; ^2^ missing = 13; ^3^ Defined as smoked regularly or occasionally in the past 12 months; * statistically significant
